# Pathway-specific protein domains are predictive for human diseases

**DOI:** 10.1371/journal.pcbi.1007052

**Published:** 2019-05-10

**Authors:** Jung Eun Shim, Ji Hyun Kim, Junha Shin, Ji Eun Lee, Insuk Lee

**Affiliations:** 1 Department of Biotechnology, College of Life Science and Biotechnology, Yonsei University, Seoul, Korea; 2 Yonsei Biomedical Research Institute, Yonsei University College of Medicine, Seoul, Korea; 3 Department of Health Sciences and Technology, SAIHST, Sungkyunkwan University, Seoul, Korea; 4 Samsung Biomedical Research Institute, Samsung Medical Center, Seoul, Korea; 5 Department of Biomedical Systems Informatics, Yonsei University College of Medicine, Seoul, Korea; University of Zurich and Swiss Institute of Bioinformatics, SWITZERLAND

## Abstract

Protein domains are basic functional units of proteins. Many protein domains are pervasive among diverse biological processes, yet some are associated with specific pathways. Human complex diseases are generally viewed as pathway-level disorders. Therefore, we hypothesized that pathway-specific domains could be highly informative for human diseases. To test the hypothesis, we developed a network-based scoring scheme to quantify specificity of domain-pathway associations. We first generated domain profiles for human proteins, then constructed a co-pathway protein network based on the associations between domain profiles. Based on the score, we classified human protein domains into pathway-specific domains (PSDs) and non-specific domains (NSDs). We found that PSDs contained more pathogenic variants than NSDs. PSDs were also enriched for disease-associated mutations that disrupt protein-protein interactions (PPIs) and tend to have a moderate number of domain interactions. These results suggest that mutations in PSDs are likely to disrupt within-pathway PPIs, resulting in functional failure of pathways. Finally, we demonstrated the prediction capacity of PSDs for disease-associated genes with experimental validations in zebrafish. Taken together, the network-based quantitative method of modeling domain-pathway associations presented herein suggested underlying mechanisms of how protein domains associated with specific pathways influence mutational impacts on diseases via perturbations in within-pathway PPIs, and provided a novel genomic feature for interpreting genetic variants to facilitate the discovery of human disease genes.

## Author summary

Protein domains are basic functional units of proteins, yet domain-based pathway annotations for proteins are challenging tasks because many domains are pervasive among diverse pathways. Therefore, we developed a network-based scoring scheme to measure pathway specificity of domains, and then used it to identify pathway-specific domains. Surprisingly, we observed substantially more disease mutations in pathway-specific domains than non-specific domains. We found evidences that mutations of pathway-specific domains tend to perturb pathway integrity via disrupting within-pathway protein-protein interactions. We also demonstrated prediction capacity of pathway-specific domains for complex diseases with experimental validations. Our study demonstrated the usefulness of pathway information for protein domains in interpreting non-random distribution of disease mutations among domains and identification of disease genes and variants.

## Introduction

Protein domains are the structural, evolutionary, and functional units of proteins. Because domains are the basic building block of protein structure and an evolutionary module [1] that increases the protein repertoire by duplication, recombination, and divergence [2], domain-centric annotation of proteins on function, phenotypes and diseases has been one of major research goals in computational biology [3]. A previous study reported that molecular function annotation can be transferred by sequence homology with only 35% accuracy for pairs of multi-domain proteins [4]. Given that majority of the eukaryotic proteins contain multiple domains, simple homology-based method would not provide reliable functional annotations for proteins in multi-cellular organisms including humans. Moreover, sequence-based annotation transfer is even less accurate for biological processes than for molecular functions [5]. Although biological processes and pathways are not exactly equivalent, we often refer to both as *pathways*. The lower reliability of sequence-based annotation for pathways are partly due to the fact that many domains are pervasive among diverse pathways. For example, the ‘winged helix-turn-helix DNA-binding’ domain occurs in many DNA-binding proteins that are involved in diverse pathways. Nevertheless, some domains may be associated with certain pathways with high specificity. Therefore, domain-based annotation of pathways requires a quantitative method which can incorporate not only sequence similarity but also specificity of domain-pathway associations.

Human complex diseases are generally viewed as pathway-level disorders. Given that a large portion of disease-associated genes are also strongly associated with specific pathways [6], protein domains associated with specific pathways may provide functional insights for the study of human diseases. Genome-wide investigations of disease-associated genetic variations have recently revealed many disease-associated genes. The observed associations between diseases and pathways triggered a boom in pathway-based analyses of disease-associated variants derived from genome-wide association studies (GWASs) and whole exome sequencing (WES) [7, 8]. More recently, domain-level distribution of pathogenic variants revealed high concentrations for particular domains [9–13], which implies that particular classes of domains are highly implicated in human diseases. Therefore, we hypothesized that pathway-specific domains could be highly informative for human diseases.

Here, we present a network-based scoring scheme to quantify pathway specificity of protein domains, which can be used to identify domains associated with specific pathways. We first generated domain profiles for human proteins then constructed a co-pathway protein network based on the associations between domain profiles. Based on the score, we classified human protein domains into pathway-specific domains (PSDs) and non-specific domains (NSDs). Interestingly, we observed a significant enrichment of disease-associated mutations for PSDs, where mutations tend to disrupt interfacing domains that mediate within-pathway protein-protein interactions (PPIs) and to have a moderate number of domain interactions. These results suggest that mutations in PSDs are likely to disrupt within-pathway PPIs, resulting in pathway disorders. Finally, we demonstrated the utility of pathway-specific domains in predicting disease-associated genes with experimental validations in zebrafish.

## Results

### Identification of pathway-specific domains (PSDs)

**Fig 1:**
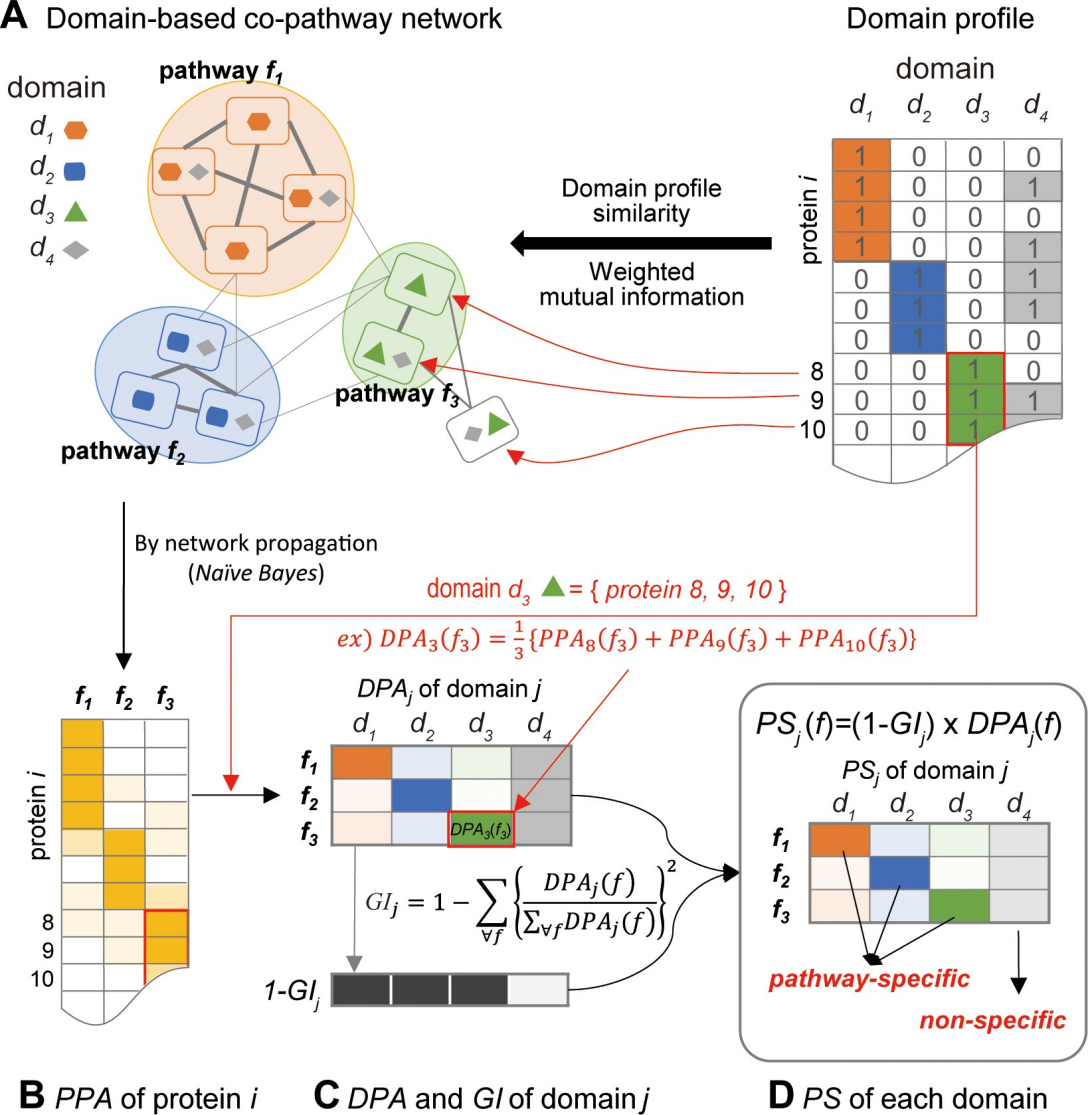
Overview of scoring pathway specificity of the protein domains. **(A)** A co-pathway protein network was constructed based on similarity of the protein domain profiles (0 and 1 represent absence and presence of each domain, respectively, in the protein). Sub-networks that represent pathway *f*_*1*_, *f*_*2*_, and *f*_*3*_ were enriched for domain *d*_*1*_, *d*_*2*_, and *d*_*3*_, respectively. Probability operating the same pathway is proportional to the edge thickness. **(B)** Next, each protein received a protein-pathway association (*PPA*) score for a specific pathway *f* by sum of edge scores to all member proteins of the pathway *f*. **(C)** Domain-pathway association (*DPA*) score of each domain was assigned by the average *PPA* of all proteins that harbor the domain. In this example, *DPA* of domain *d*_*3*_ for pathway *f*_*3*_, *DPA*_*3*_(*f*_*3*_), was assigned by the average of *PPA*_*8*_(*f*_*3*_), *PPA*_*9*_(*f*_*3*_), and *PPA*_*10*_(*f*_*3*_). Gini Index (*GI*) was used to measure the impurity of the data. **(D)** Subsequently, pathway specificity (*PS*) was calculated. In this example, because domain *d*_*1*_, *d*_*2*_, and *d*_*3*_ have high *PS*s for pathway *f*_*1*_, *f*_*2*_, and *f*_*3*_, respectively, they were classified as pathway-specific domains (PSDs) for the corresponding pathways. However, domain *d*_*4*_ was classified as a non-specific domain (NSD) due to the low *PS* for all pathways.

We previously found that human protein interactions can be accurately retrieved by associations between domain profiles with a scoring scheme based on information theory, *WMI*, which assigns more weight to rarer domains in calculating the *MI* [14]. The resultant domain-based network (**Fig 1A**) was highly predictive for proteins that operate the same human GOBP pathways. Using a Bayesian statistics framework, we assigned *LLS*s [15] to the links of the co-pathway network.

Because the network links were based on domain-sharing patterns among proteins for the same pathway, domains enriched for a pathway also likely connect to other proteins for the same pathway (**Fig 1B**). We therefore measured domain-pathway associations (**Fig 1C**) based on the network connections from a domain to the member proteins of the pathway. However, overall strength of the network connections for a domain-pathway association does not guarantee their specificity. We thus accounted for the distribution of each domain across pathways using the Gini Index (*GI*). In summary, the network-based scoring scheme *PS* quantifies pathway-specific associations for each protein domain (**Fig 1D**).

**Fig 2:**
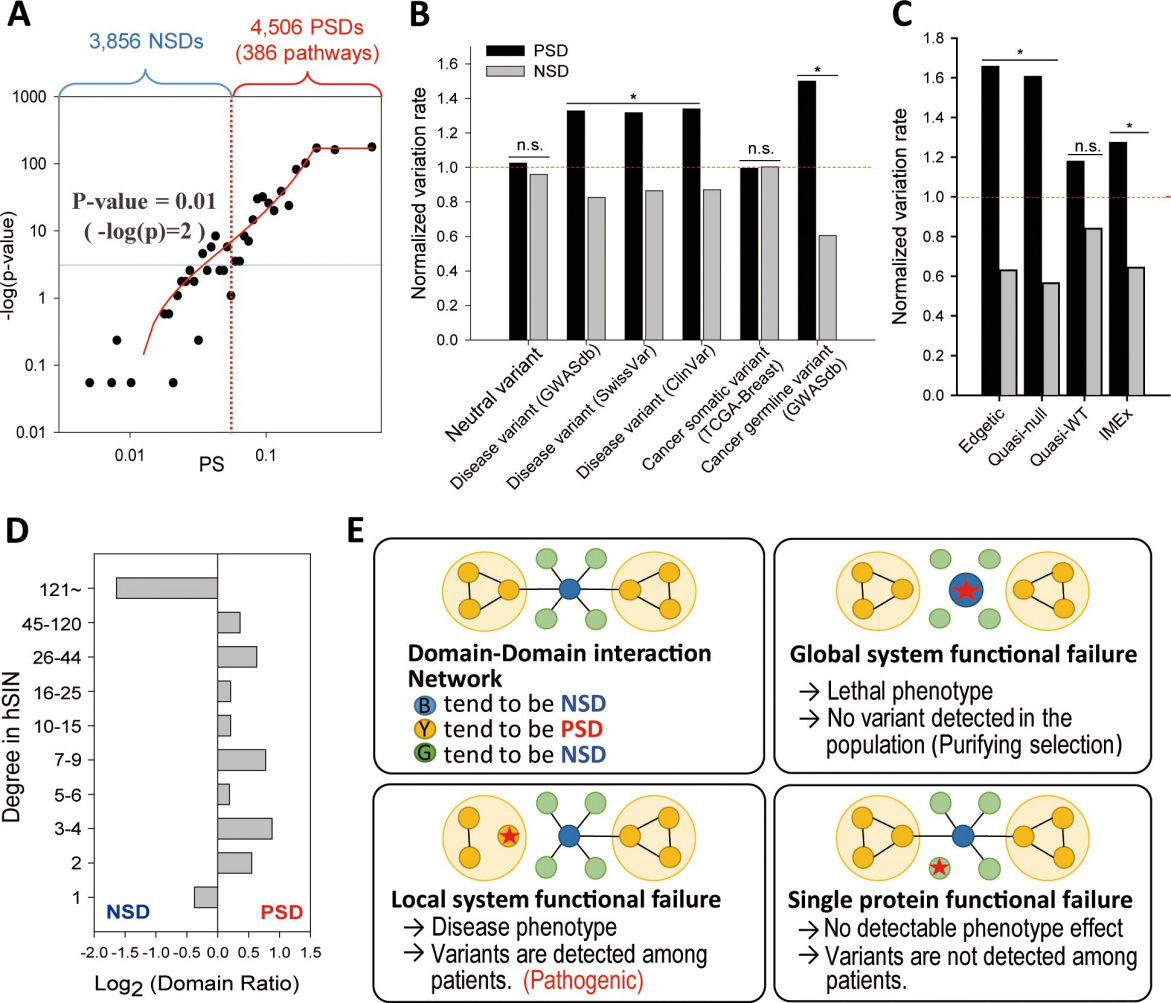
Disease implications of PSDs. **(A)** Regression between pathway specificity (*PS*) and the significance of overlap with the gold-standard domain-pathway pairs by sigmoidal curve fitting. Domain-pathway associations were divided into two groups: the top 16,000 associations that showed significant overlap (*p* < 0.01 by Fisher’s exact test) with the gold-standard data, and the remaining 33,636 associations. 4,506 domains for the top 16,000 associations were defined as pathway-specific domains (PSDs) and 3,856 domains for the remaining associations were defined as non-specific domains (NSDs). **(B)** Comparison of normalized variation rates (NVRs) for neutral and pathogenic variants between PSDs and NSDs (*, *P* < 0.01; n.s., *P* > 0.05) **(C)** Comparison of NVRs for three classes of missense disease mutations described by Sahni *et al*. and nonsynonymous variants known to affect physical protein interactions by IMEx consortium between PSDs and NSDs (*, *P* < 0.01; n.s., *P* > 0.05). **(D)** Comparison of the ratios (log base 2) of PSDs to NSDs for groups of human structural interaction network (hSIN) interfacing domains with similar sizes for different ranges of domain interaction connectivity. **(E)** Proposed models for the relationships between mutational consequences and the number of domain interactions. The blue node represents a hub domain that mediates interactions between a large number of proteins that contain domains with a single or a few, at most, interacting domains (green nodes), and the yellow nodes represent domains with moderate numbers of domain interactions, which are involved in ‘within-pathways’ (shaded areas).

We calculated *PS* of human protein domains derived from the InterPro database for GOBP pathways. To assess accuracy of domain-pathway associations, we compiled gold-standard domain-pathway associations between InterPro domains and GOBP pathways derived from InterPro2GO [16] annotations, as these are based on manual curation. We found that only 27% of InterPro domains have annotated GOBP terms by InterPro2GO. We observed strong positive correlations between *PS* and the likelihood of gold-standard domain-pathway associations, in which approximately 16,000 associations between 4,506 InterPro domains and 386 GOBP pathways were more than twice as likely to be gold-standard associations as would be expected by random chance (**S1 Fig**). The significance of the agreements with gold-standard associations was also assessed by Fisher’s exact test. We observed similarly high correlations between *PS* and gold-standard data, where the top 16,000 domain-pathway associations significantly overlapped (*P* ≤ 0.01) with gold-standard data (**Fig 2A**). We defined 4,506 InterPro domains from the top 16,000 significant domain-pathway associations as pathway-specific domains (PSDs) and the remaining 3,856 InterPro domains as non-specific domains (NSDs). The *PS* threshold for the division between the two domain classes (*P* ≤ 0.01) was 0.056.

### PSDs are enriched for disease-associated variants

Recently, investigations into the protein domain-level landscapes of cancer somatic mutations have revealed domains that are enriched for somatic and germline mutations, and domain-level mutational hot spots, which facilitate the identification of novel cancer genes and functional mutations, and provide mechanistic insights regarding mutational consequences [9–13]. To investigate the relationship between disease susceptibility and pathway specificity at the domain level, we compared the frequencies of disease-associated germline variants between PSDs and NSDs derived from the following databases: GWASdb [17], a database of human genetic variants from GWASs; SwissVar [18]; and ClinVar [19]. We calculated the normalized variation rate (*NVR*), which represents the probability of a variant occurring in a PSD or NSD normalized to the number of variants observed in both types of domains. Notably, we observed an approximately 1.5-fold higher *NVR* in PSDs than in NSDs for all three pathogenic variant sets (**Fig 2B**). We found that the observed enrichment of PSD for disease-associated variants was not significantly affected by moderate changes in *PS* threshold for defining PSDs (**S3A Fig**). In contrast, variants with neutral effects derived from the HumVar neutral training set for PolyPhen-2 [20] exhibited similar *NVR*s between PSDs and NSDs. We also performed a similar analysis using cancer somatic mutations from the TCGA for several cancer types including breast cancer [21], and found no significant differences in *NVR*s between PSDs and NSDs. Notably, germline cancer variants from the GWASdb set exhibited an approximately 1.5-fold higher *NVR* for PSDs than for NSDs (1.5 for PSDs and 0.6 for NSDs). These results indicate that PSDs are more susceptible to the diseases by inherited genetic variants, but not by somatic mutations.

### PSD mutations are likely to disrupt within-pathway PPIs

To provide mechanistic insight for the higher frequency of pathogenic variants in PSDs than in NSDs, we investigated the relationship among the disease-associated mutations, the pathway specificity of the domains, and the domain-level interaction network. Our analysis was motivated by the following three recent observations: (i) the majority of disease-associated variants exert pathogenic effects via perturbations of PPIs rather than on protein folding or stability [22]; (ii) disease-associated variants are enriched in PPI-interfacing domains [23–25]; and (iii) the majority of disease genes are not essential and do not encode hub proteins [26].

For example, a recent large-scale characterization of disease-associated mutations revealed that most missense disease mutations do not severely altered protein structure or stability, but rather that they tend to perturb PPIs in the majority of the wildtype proteins [22]. In the study, missense disease mutations were divided into three classes by the effects on molecular interactions or “edgotype” [27]: no apparent detectable change in interactins (“quasi-WT”), partial loss of interactions (“edgetic”), and apparent complete loss of interactions (“quasi-null”). Importantly, two-thirds of the tested disease mutations belonged to the edgetic or quasi-null classes. These observations suggest that many mutation-disease associations may be understood via mutational effects on PPIs. Thus, we compared the frequencies of each disease mutation class between PSDs and NSDs and found that edgetic and quasi-null disease mutations exhibited >2.5-fold higher *NVR* for PSDs than for NSDs (**Fig 2C**). We also found approximately 2-fold enrichment of PSD for nonsynonymous variants affecting on physical protein interactions recently published by IMEx consortium [28]. We confirmed that moderate changes in *PS* threshold for defining PSDs did not significantly influence enrichment of PSD for nonsynonymous variants affecting on physical protein interactions by IMEx (**S3B Fig**). In contrast, the *NVR* of the quasi-WT class of mutations was approximately 1.5-fold higher for PSDs than for NSDs, which was similar to the fold change for other disease-associated variant sets (see **Fig 2B**), indicating that PSDs are enriched for disease mutations that cause loss of wildtype PPIs. PPIs are mediated by domain-level interactions. Therefore, these results suggest that PSDs are more important than NSDs for PPIs, whose perturbations can result in phenotypic changes.

To further investigate the impact of PSDs on phenotype via PPIs, we next performed domain-level network analyses based on the human structural interaction network (hSIN) [25], which mapped 135,166 interactions between 590 interfacing domains, of which 345 and 245 were PSDs and NSDs, respectively. We compared the ratios (log base 2) of PSDs to NSDs for groups of human structural interaction network (hSIN) interfacing domains for different ranges of domain interaction connectivity. Given that domain-level network degree is not evenly distributed, we defined groups of domains for comparisons not by equal degree interval but by similar group size. We found that PSD is enriched—indicated by positive log_2_(Domain Ratio) score—for interfacing domains with a moderate number (2–120) of domain interactions, whereas PSD is depleted—indicated by negative log_2_(Domain Ratio) score—among interfacing domains with either a single interaction or more than 121 interactions (**Fig 2D**). To explain the observed higher frequency of PSDs among interfacing domains with a moderate number of domain interactions, we proposed a model of mutational consequences via the disruption of interfacing domains with different degrees of connectivity (**Fig 2E**). Mutations on interfacing domains with a single protein interaction (green nodes) may result in the functional failure of a single protein and no detectable pathogenic effect. Consequently, these mutations are not detected among patients. If mutations occur in a hub-interfacing domain (blue node), the interactions toward many proteins involved in diverse pathways may be disrupted, which may result in the functional failure of the global system. In this case, mutations would generally cause lethal phenotypes, resulting in purifying selection of the mutation. In contrast, mutations on interfacing domains with a moderate number of domain interactions (yellow nodes), which likely corresponds to the range of the pathway size, disrupt the interactions of proteins within that pathway, which may result in the functional failure of local systems. Because the majority of disease genes are enriched for pathways [6], these mutations are likely to cause the functional failure of disease-associated pathways, and can be detected in patients. Therefore, the higher frequency of PSDs among domains with a moderate number of domain interactions suggests that PSDs are more likely involved in heritable diseases via mutations that disrupt within-pathway PPIs.

### PSDs are predictive features for human diseases

**Fig 3:**
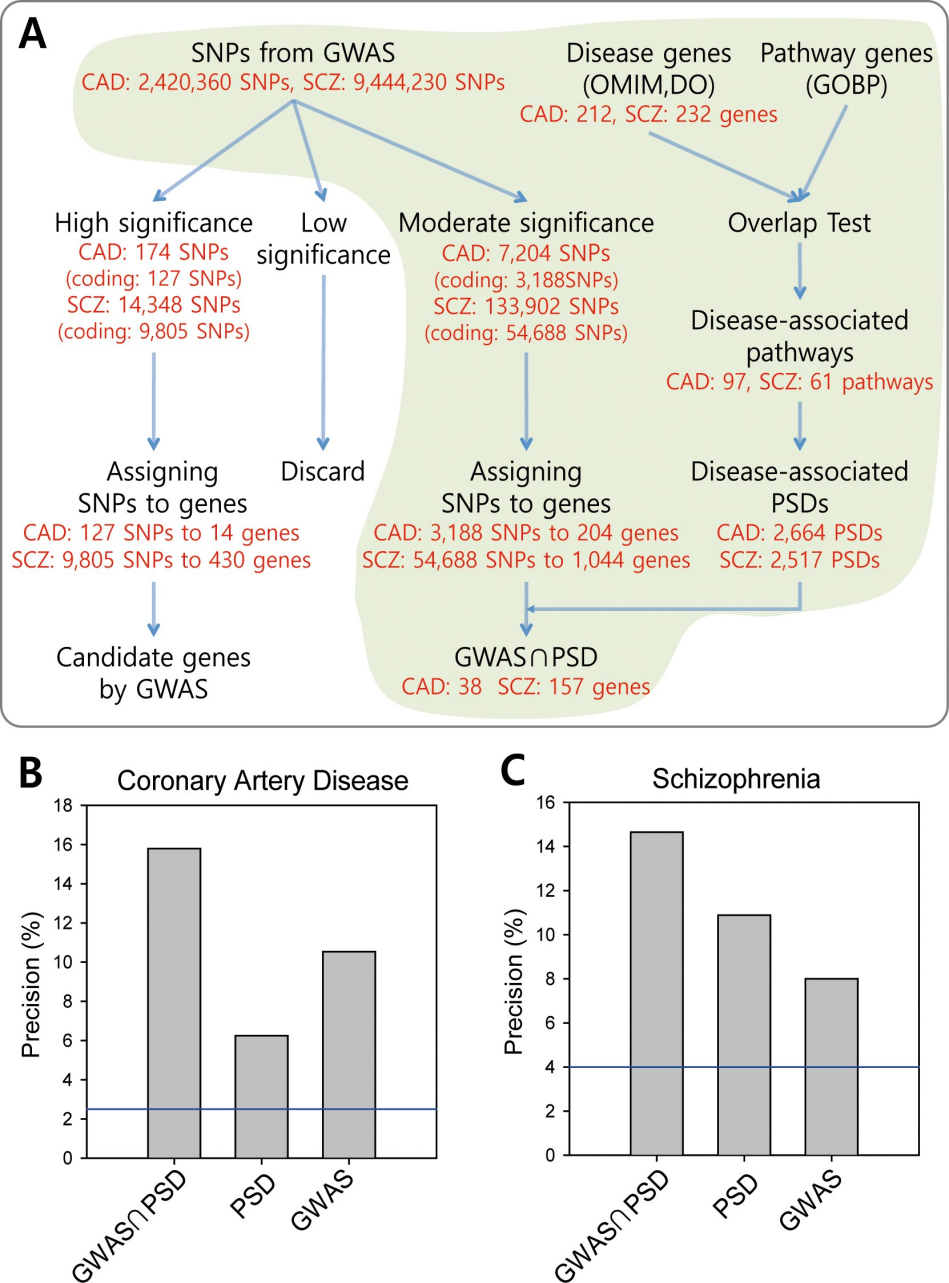
PSDs can predict disease genes. **(A)** A summary of candidate gene selection for coronary artery disease (CAD) and schizophrenia (SCZ) by integration of GWAS significance and PSD occurrence data. SNPs from GWASs were divided into three groups: (i) SNPs with high significance that indicate confident candidate genes; (ii) SNPs with low significance that are generally discarded; and (iii) SNPs with moderate significance that were considered for further selection in this study. Based on the overlap between disease genes and pathway genes, we converted domain-pathway associations into domain-disease associations to identify disease-associated PSDs. Candidate disease genes of the GWAS∩PSD set were selected based on the occurrence of disease-associated PSDs of the genes with moderate GWAS significance. **(B)** The precision of CAD gene predictions was assessed based on CADgeneDB annotations. The precision by random expectation (i.e., the number of disease genes / the number of all human genes) is indicated by the blue line (~2.5%). **(C)** The precision of SCZ predictions was assessed based on SZdatabase annotations. The precision by random expectation is indicated by the blue line (~4.1%).

Given that PSDs are more susceptible to the heritable diseases, we hypothesized that PSDs could be predictive genomic features for human diseases. Even their modest prediction power could be highly useful if integrated with other disease-associated genomic information. For example, GWASs generally test for associations of more than a million single-nucleotide polymorphisms (SNPs) for each disease phenotype, but identify only a few candidates due to highly stringent significance thresholds (e.g., *p* ≤ 10^−7^). However, GWASs usually detect a large number of candidate genes with moderate associations that have *p*-values above this stringent threshold (e.g. 10^−7^ < *p* ≤ 10^−3^). Additional candidate genes, i.e., those with moderate GWAS significance, may be rescued by meta-analyses with larger sample sizes, but such studies are costly to conduct. We hypothesized that an additional disease-associated feature would enable us to distinguish disease genes from non-disease genes among those with moderate GWAS significance. Therefore, we tested whether disease-associated PSDs could identify disease genes among candidates with moderate GWAS significance derived from two publicly available data sets: CARDIoGRAM [29], a study of coronary artery disease (CAD); and the Psychiatric Genomic Consortium (PGC) [30], a study of schizophrenia (SCZ) (**Fig 3A**). To conduct gene-centric analyses, we identified SNPs with moderate GWAS significance that were located within 10 kb upstream or downstream of the gene, resulting in 204 and 1,044 genes moderately associated with CAD and SCZ, respectively. We then identified PSDs associated with CAD or SCZ. PSD-pathway relationships were converted into PSD-disease relationships based on significant overlap (*P* < 0.01 by Fisher’s exact test) between disease-associated genes and pathway-associated genes. We compiled 212 disease-associated genes for CAD and 233 disease-associated genes for SCZ from OMIM [31] and DO [32]. Based on Fisher’s exact test (*p* < 0.01), we identified 2,664 PSDs for CAD (**S2 Table**) via 97 CAD-associated GOBP pathways (**S3 Table**), and 2,517 PSDs for SCZ (**S4 Table**) via 61 SCZ-associated GOBP pathways (**S5 Table**). For GOBP pathways, we considered only those that contained at least five member genes. We further selected CAD and SCZ candidate genes with moderate GWAS significance based on the number of disease-associated PSDs in each gene (S6 and S7 Tables). We selected candidate genes with moderate GWAS significance in which at least three disease-associated PSDs occurred (GWAS∩PSD set), resulting in 38 genes for CAD and 157 genes for SCZ.

Next, disease predictions made by PSDs were validated using independent disease annotations from two disease-specific databases: 604 CAD-associated genes from CADgene V2.0 [33] and 936 SCZ-associated genes from SZdatabase [34]. To further ensure the independence of the validation gene set, we excluded 212 CAD genes and 233 SCZ genes that overlapped with genes that were used to identify disease-associated PSDs, resulting in 466 CAD genes and 767 SCZ genes for the final validation sets. To compare the predictions with GWAS significance only or PSD significance only, we also prepared similar sets that included predictions based on *p*-values among genes with moderate GWAS significance (GWAS set) and on the number of disease-associated PSDs among genes with both moderate and low GWAS significance (PSD set). A clear benefit of using PSDs was observed for CAD, as approximately 30% more CAD genes were identified in the GWAS∩PSD set than in the GWAS set (**Fig 3B**). An even greater benefit was observed for SCZ (**Fig 3C**). Interestingly, the PSD set was more predictive for SCZ than the GWAS set. For both CAD and SCZ, the combination of GWASs and PSDs outperformed GWASs only and PSDs only, indicating that GWASs and PSDs contributed largely complementary information about the diseases.

### PSD-based identification of novel heart disease genes with experimental validation in zebrafish

**Fig 4:**
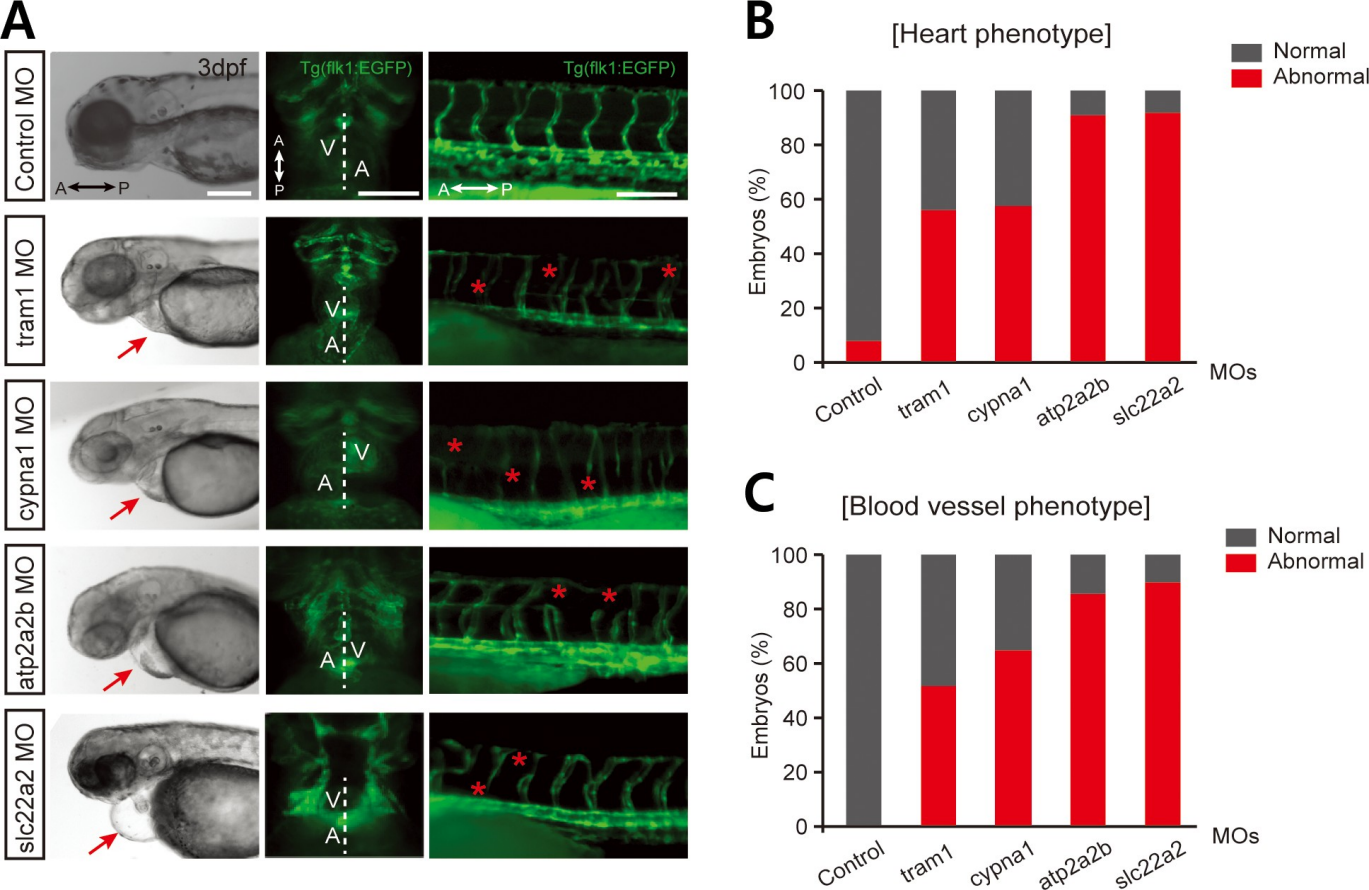
Experimental validation of novel genes for heart development in zebrafish. **(A)** Tg(*flk1*:*EGFP*) zebrafish embryos injected with morpholinos (MOs) for novel candidate genes for CAD showed morphological heart abnormalities, such as peripheral edema at 3 days post-fertilization (arrows in the left panel, scale bar = 500 μm). Zebrafish embryos normally have hearts with a left ventricle (V) and right atrium (A), whereas the embryos injected with MOs related to CAD genes exhibited either no asymmetry or reversed V and A orientation (middle panels, scale bar = 200 μm). These embryos also exhibited malformed blood vessels in the trunk (asterisks in the right panel, scale bar = 200 μm). **(B)** MO-injected Tg(*flk1*:*EGFP*) zebrafish embryos were counted to quantify those that exhibited heart asymmetry. **(C)** MO-injected Tg(*flk1*:*EGFP*) zebrafish embryos were counted to quantify those that exhibited vascular defects. Over 20 MO-injected embryos per gene were counted for each analysis **(A-C)**.

Next, we experimentally validated the predictions of a GWAS∩PSD set using a morpholino-based loss-of-function phenotype analysis in zebrafish. Although the majority of human disease genes have zebrafish orthologs [35], some disease phenotypes, such as those of psychiatric diseases, are not readily classified in zebrafish. Therefore, we tested predictions for CAD genes only. We found 23 zebrafish orthologs for the 38 human candidate CAD genes from the GWAS∩PSD set. After excluding genes that were already known to be involved in CAD or that were highly ranked by GWAS, we selected the following four testable candidate genes in zebrafish for further analysis: *tram1*, *apod*, *cypna1*, and *slc22a2*. Unfortunately, the zebrafish model for CAD has not been well established. However, we found that 207 human orthologs of zebrafish genes for heart or blood vessel development by GO annotations were significantly associated with CAD genes indicated by OMIM or DO annotations (*p* < 1.29 × 10^−4^, Fisher’s exact test) or by CADgeneDB annotations (*p* < 7.46 × 10^−3^, Fisher’s exact test), indicating significant associations between CAD and heart/vessel development at the pathway level. These results indicate that zebrafish genes validated by abnormal heart or blood vessel phenotypes during embryonic development may have implications for human CAD. To confirm the feasibility of CAD gene validation based on heart/vessel phenotype, *atp2a2b*, which has been implicated in CAD [36], was included as a positive control. After microinjection of test gene morpholinos into zebrafish embryos, heart and blood vessel phenotypes were examined using a fluorescent stereomicroscope (**Fig 4A** and **S2 Fig**). We found that the majority of embryos with morpholino injections exhibited abnormal heart or blood vessel phenotypes, not only in the CAD-associated *atp2a2b* group, but also in three of the four candidate gene groups, including the *tram1*, *cypna1*, and *slc22a2* groups (**Fig 4B and 4C**), strongly implicating the association of these genes with CAD.

## Discussion

The network-based quantitative method of modeling domain-pathway associations presented herein suggested underlying mechanisms of how protein domains associated with specific pathways influence mutational impacts on diseases via perturbations in within-pathway PPIs, and provided a novel genomic feature for interpreting genetic variants to facilitate the discovery of human disease genes.

Stratification of coding regions by different susceptibilities to heritable pathogenic variations may improve the assessment of genomic risk for complex human diseases based on exonic variations. For example, if we can identify PSDs for a particular disease as described in this work, more weight may be assigned to the mutations located in the PSDs for the disease than those located in the NSDs in assessing disease risk. Additionally, disease-associated PSDs would be useful predictors for disease gene candidates. The insufficient statistical power of GWASs often omits a large number of SNPs with moderately significant disease associations. Thus, in theory, we may apply the demonstrated procedure of candidate gene selection with moderate significance based on disease-associated PSDs to all GWASs, which may reveal many disease gene candidates that are missed during conventional GWAS analyses. Therefore, PSDs will significantly contribute to the genetic dissection of human diseases.

In this study, we present a scoring scheme *PS*, which quantifies specificity of domain-pathway associations. Although the given quantification strategy was demonstrated in human only, its application to other organisms is conceptually straightforward: (i) generate domain profiles for proteins using InterPro databases, (ii) construct a co-pathway protein network based on the associations between domain profiles, (iii) calculate *PS* and identify PSDs using gold-standard domain-pathway pairs, (iv) infer GOBP pathway terms of proteins or predict proteins with phenotypic effects using the identified PSDs.

This network-based scoring scheme to quantify specificity of domain-pathway associations may be a significant addition to our current computational tool box for pathway annotation of domains and proteins. For example, *PS* can prioritize GOBP terms for an InterPro domain, which may facilitate manual curation for novel entries in the InterPro2GO database. Furthermore, probabilistic models of pathway involvement of proteins could be developed based on *PS*.

## Methods

### Generation of domain profiles

Information regarding domain occurrence for human proteins was downloaded using the BioMart search tool (http://www.ensembl.org/biomart/martview) from the InterPro [37] database (v38). We generated domain profiles, which were represented as an array of Boolean values for each protein with 1 and 0 indicating the presence and absence of a given domain in the protein, respectively. We generated domain profiles for 17,013 human protein coding genes using a total of 8,362 InterPro domains.

### Construction of a domain-based co-pathway network

A co-pathway protein network was constructed based on the association between domain profiles as described in our previous study [14] and summarized as follows. Most domain profiles are sparse, because most proteins have few domains only. Domain profiles for proteins with more complex domain compositions were considered more informative than those with simpler compositions. To take into account the non-uniformity of information across profiles, we employed mutual information (*MI*), which considers the entropy (i.e. complexity) of profiles. The *MI* does not require an a priori model, and has high robustness and accuracy for a wide variety of applications. Additionally, the amount of information across individual domains seemed to vary. We observed a power-law distribution of domain occurrence among proteins, from which we hypothesized that rare domains were associated with relatively specific biological processes and prevalent domains contributed to diverse functions. Therefore, we assigned higher weights to rarer domains during the *MI* calculation, resulting in weighted mutual information (*WMI*). The weight for each domain was calculated as described in the following definitions.

**Definition 1.** Domain-specific weight *𝜔*_*j*_

Given *n* proteins and *m* domains, the domain-specific weight *𝜔*_*j*_ for each domain *j* (1 ≤ *j* ≤ m) is defined as:

$$\omega_{j}=\frac{\sum_{k=1}^{n}\sum_{l=1}^{m}c_{kl}}{\sum_{k=1}^{n}c_{kj}}$$

where *c*_*kl*_ represents the occurrence value (0 or 1), assigned by whether *k*^*th*^ protein contains *l*^*th*^ domain.

**Definition 2.** Weighted mutual information *I*_ω_

Given two proteins *X* and *Y*,

$$I_{\omega }(X,Y)=H_{\omega }(X)+H_{\omega }(Y)-H_{\omega }(X,Y)$$

where *H*_*ω*_(*X*) and *H*_*ω*_(*Y*) represent the weighted entropy of protein *X* and protein *Y*, respectively, and can be calculated as follows:

$$H_{\omega }(X)=-\sum_{t\in \{0,1\}}\left\{p_{\omega }(X,t)∙\log\;p_{\omega }(X,t)\right\},\;p_{\omega }(X,t)=\frac{\sum_{j\in \{j|c_{Xj}=t\}}\omega_{j}}{\sum_{j=1}^{m}\omega_{j}}$$

where *t* represents domain profile value of protein *X*, which can be {0,1} because we adopt Boolean domain profile.

Additionally, *H*_*ω*_(*X*,*Y*) represents the weighted joint entropy between *X* and *Y*, and can be calculated as follows:

$$H_{\omega }(X,Y)=-\sum_{t_{1}t_{2}\in \{(00,01,10,11\}}\left\{p_{\omega }(XY,t_{1}t_{2})∙log\;p_{\omega }(XY,t_{1}t_{2})\right\},$$

$$p_{\omega }(XY,t_{1}t_{2})=\frac{\sum_{j\in \{j|c_{Xj}\;is\;t_{1}\;and\;c_{Yj}\;is\;t_{2}\}}\omega_{j}}{\sum_{j=1}^{m}\omega_{j}}$$

where *t*_*1*_ and *t*_*2*_ represents domain profile value of protein *X* and protein *Y*, respectively.

### Log likelihood score

The weight of a protein-protein link or a domain-pathway link was measured by a log likelihood score (*LLS*), which was based on a Bayesian statistical framework as previously described [15].

**Definition 3.** Log likelihood score of a protein-protein link or a domain-pathway link

$$LLS=ln\left(\frac{P(L|E)/P(⌐L|E)}{P(L)/P(⌐L)}\right),if\;P(L)\neq 0\;and\;P(⌐L|E)\neq 0$$

where *P*(*L*|*E*) and *P*(⌐*L*|*E*) represent the frequencies of positive (*L*) and negative (⌐*L*) gold-standard links observed in the given evidences (*E*), while *P*(*L*) and *P*(⌐*L*) represent the prior expectations (i.e. the total frequencies of all positive and negative gold-standard links, respectively). In Bayesian words, *P*(*L*)/*P*(⌐*L*) is *prior odds* and *P*(*L*|*E*)/*P*(⌐*L*|*E*) is *posterior odds*. The *posterior odds* are the *prior odds* times the Bayes factor, likelihood. For protein-protein links, we first sorted them by confidence score (e.g. *WMI*), then computed *LLS* for each bin of 1000 protein pairs. For the given size of binning, we hardly encountered with *P*(*L*) = 0 *or P*(⌐*L*|*E*) = 0. However, if so, we could avoid the problem by taking larger bin size. Protein-protein pairs or domain-pathway pairs with positive *LLS* values are more likely to be associated with each other for the given evidence than those by random chance. For this study, the positive gold-standard protein-protein links were generated by pairing two proteins annotated for the same GOBP terms [38] and negative gold-standard protein-protein links were generated by pairing two proteins annotated for different GOBP terms. The positive gold-standard domain-pathway links were compiled from the InterPro2GO database and negative gold-standard domain-pathway links were generated by pairing a domain and a pathway that belong to the database but are not associated with each other.

### Pathway specificity (*PS*) score

We developed a metric, Pathway Specificity (*PS*), to quantify the specificity of domain-pathway associations, based on the combination of connectivity from a domain to the member proteins of the pathway and domain-specific weights. For the computing *PS*, we defined a domain-based co-pathway network by taking protein-protein links with only positive *LLS*. As a first step in the *PS* calculation, the protein-pathway association (*PPA*) score of each protein for a specific pathway was calculated via summation of *LLS*s to the protein in the domain-based co-pathway network. Subsequently, we transformed the association score based on the *LLS* into the probability score. We observed that the sum of *LLS*s of protein pairs followed a power-law distribution. Thus, we modeled the sum of the *LLS*s as a Pareto distribution, which is a power-law probability distribution that coincides with social, scientific, geophysical, and many other types of observable phenomena. The *p*-value of the Pareto distribution is calculated as follows:

$$P_{Pareto}(X>x_{i})={\left(\frac{x_{min}}{x_{i}}\right)}^{\alpha }$$

where *x*_*i*_ is sum of *LLS* of protein *i* and *x*_*min*_ is the scale parameter empirically plugged in by the minimum of sum of *LLS* values and *α* is the shape parameter that determines the steepness of the slope. As *α* increases, the *p*-value of the Pareto distribution is exponentially distributed with intensity *α*. We wanted to reduce the skewness of sum of *LLS* distribution by transformation into *P*_*Pareto*_, which is subsequently used to compute pathway association of each protein. The number of proteins with sum of *LLS* is subject to the size of pathways. If a pathway has a small number of member proteins, *α* tends to be very small. We found that if *α < 1*, the skewness of the sum of *LLS* distribution did not significantly improved. Therefore, we empirically take *1* as the minimum value of *α* to calculate protein-pathway association score as in **Definition 4**.

**Definition 4.** Protein-pathway association (*PPA*) score for a specific pathway *f*

For a given protein *i*, the *PPA* score *PPA*_*i*_ (*f*) is defined as follows:

$${PPA}_{i}(f)=1-P_{Pareto}(X>s_{i}(f))=1-{\left(\frac{s_{min}(f)}{s_{i}(f)}\right)}^{\alpha (f)}$$

$$\left\{ \begin{array}{l} \mathrm{scale}\;\mathrm{parameter}\;s_{min}(f):\;\mathrm{minimum}\;\mathrm{value}\;ofs_{i}(f)\;\mathrm{for}\;a\;\mathrm{specific}\;\mathrm{pathway}\;f\\ \;\mathrm{shape}\;\mathrm{parameter}\;\alpha (f)=1+n{\left[\sum_{i=1}^{n}\ln\left(\frac{s_{i}(f)}{s_{min}(f)}\right)\right]}^{-1} \end{array}\right.$$

Here, *s*_*i*_ (*f*) was calculated via summation of the *LLS*s as follows:

$$s_{i}(f)=\sum_{k=\{x|x\in G_{i}\;and\;F\}}{lls}_{ik},$$

where *lls*_*ik*_ denotes the *LLS* of a link between protein *i* and protein *k*, *G*_*i*_ indicates a set of all proteins connected to gene *i* in the network, and *F* indicates a set of proteins annotated for pathway *f*. We have assigned probability scores on edges of protein-protein interaction network using Pareto distribution. The *s*_*i*_(*f*) for each protein was calculated based on degree of association to each pathway by summation of the assigned probability scores of all links to known proteins for the pathway. Using the *PPA* score and domain profile matrix, we then defined the domain-pathway association (*DPA*) score as in **Definition 5**.

**Definition 5**. Domain-pathway association (*DPA*) score for a specific pathway *f*

Given a specific domain *j*, the *DPA* score *DPA*_*j*_ (*f*) is defined as follows:

$${DPA}_{j}(f)=\frac{1}{\left|K\right|}\sum_{i\in K}{PPA}_{i}(f)∙c_{ij}$$

$$\left\{ \begin{array}{c} K:a\;set\;of\;proteins\;containing\;domain\;j\\ |K|:size\;of\;the\;set\;K\\ c_{ij}:\;occurrence\;value\;of\;the\;i^{th}\;protein\;and\;the\;j^{th}\;domain,\;mentioned\;in\;Definition\;1\\ {PPA}_{i}(f):\;PPA\;score\;of\;protein\;i\;for\;a\;specific\;pathway\;f \end{array}\right.$$

Then, we finally calculate *PS* as described in **Definition 6**.

**Definition 6.** Pathway Specificity (*PS*) for a domain *j* and a pathway *f*

$${PS}_{j}(f)=(1-{GI}_{j})\times {DPA}_{j}(f),$$

where *DPA*_*j*_(*f*) is the association score of domain *j* for a specific pathway *f*, and *GI*_*j*_ is the Gini Index of a domain *j* over all pathways, and is defined as following:

$${GI}_{j}=1-\sum_{\forall f}{\left\{\frac{{DPA}_{j}(f)}{\sum_{\forall f}{DPA}_{j}(f)}\right\}}^{2}$$

*GI*, which is a common impurity measure for classification-type problems, is maximized when the *DPA* of a domain for all pathways are equal, and is equal to zero when a domain has a *DPA* for only one pathway.

### Classification between pathway-specific domain (PSD) and non-specific domain (NSD)

We calculated *PS*s for a total of 49,636 domain-pathway associations between 5,253 InterPro domains and 407 GOBP pathways (**S1 Table**). Using manually curated associations between InterPro domains and GOBP pathways derived from InterPro2GO [16] as gold standard data, we measured likelihood of true domain-pathway association for given *PS* scores. We observed strong positive correlations between *PS* and the likelihood of gold standard domain-pathway association. The significance of the agreements with gold-standard associations was also assessed by Fisher’s exact test. We divided domain-pathway associations into two classes by high significance of agreement with gold-standard associations (*P* ≤ 0.01), by which 4,506 InterPro domains from the top 16,000 significant domain-pathway associations were defined as pathway-specific domains (PSDs) and the remaining 3,856 InterPro domains as non-specific domains (NSDs).

### Analysis of disease-associated variants

We compared the occurrence of disease-associated variants between PSDs and NSDs using pathogenic germline variants compiled from three independent sources: (i) SNPs from the GWASdb [17]; (ii) OMIM disease gene variants [31, 39]; and (iii) variants from the ClinVar database [19]. We mapped protein domain regions in the human genome using Ensembl-API. We compiled SNPs that were significantly (*p* < 10^−7^) associated with nearly 1,610 GWAS traits from GWASdb, which mapped them to dbSNP Build 142 and Genome Assembly, GRCh37/hg19, resulting in 26,342 disease-associated SNPs. Only 966 of these SNPs (~3.6%) were located in the protein-coding regions, and of these, 569 SNPs were located in InterPro domain regions. For the analysis of cancer germline variants, we compiled 51 germline variants associated with cancer studies from GWASdb, and 20,945 somatic variants from breast cancer patients from The Cancer Genome Atlas (TCGA) consortium. We also compiled 1,779 and 10,778 variants for OMIM disease genes from SwissVar (http://swissvar.expasy.org) and dbSNP (http://www.ncbi.nlm.nih.gov/snp, OmimVarLocusIdSNP.bcp file), respectively, to generate an OMIMVar set of 11,024 OMIM disease-associated variants. We found that 9,050 of these variants were located in protein domain regions. ClinVar is another major public archive of relationships among human variants and phenotypes. We obtained 13,465 ClinVar variants for the clinical significance term of ‘pathogenic’, and found that 10,680 of them were located in protein domain regions. To generate the null model, we employed variants expected to have a neutral effect, which were derived from the HumVar neutral training set from Polyphen-2 [20]. The HumVar neutral training set was constructed of common human nsSNPs (minor allele frequency > 1%) without annotated involvement in disease, which were considered to be non-damaging variants. Three classes of missense disease mutations were designated as described by Sahni *et al*. [22]: (i) quasi-WT that shows no change in wildtype interactions, (ii) edgetic that shows loss of some wildtype interactions, (iii) quasi-null that shows complete loss of wildtype interactions. We used 40, 27, and 32 missense mutations located in PSDs and 24, 15, and 36 missense mutations located in NSDs for the edgetic, quasi-null, and quasi-WT classes, respectively.

To compare occurrence of neutral or disease-associated variants between PSDs and NSDs, the total number of variants in the entire genomic region for each domain class, i.e. the variation rate (*VR*), was calculated as follows:

$$VR=\frac{\#of\;test\;variants\;for\;the\;given\;domain\;region\;(PSD\;or\;NSD)}{Total\#of\;nucleotides\;for\;the\;given\;domain\;region\;(PSD\;or\;NSD)}$$

The background variation rate (*BVR*) for all domain regions, including both PSDs and NSDs, was calculated as follows:

$$BVR=\frac{\#of\;test\;variants\;for\;all\;domain\;regions\;(PSDs\;and\;NSDs)}{Total\#of\;nucleotides\;for\;all\;domain\;regions\;(PSDs\;and\;NSDs)}$$

*VR*s for the test variant sets were then normalized to the background variation rate (*BVR*) to calculate the normalized variation rate (*NVR*) as follows:

$$NVR=\frac{VR}{BVR}$$

Statistical significance of *NVR* differences between PSDs and NSDs were evaluated by binomial tests.

### Domain-level network analysis

Wang *et al*. [25] provided information on 590 interfacing domains (IFD) and 135,166 domain-domain interactions in a structural level human protein interaction network (hSIN). Among the 590 interfacing domains, 345 domains were PSDs and 245 domains were NSDs. Therefore, there were ~1.4-fold more PSDs than NSDs. To evaluate the difference in IFD enrichment between the two domain groups, we measured the ratio of PSDs to NSDs, i.e. the domain ratio, for several groups of IFDs with different numbers of domain interactions (i.e. domain interaction connectivity) as follows:

$$Domain\;Ratio=\frac{\left|PSD\cap IFD\right|}{\left|PSD\right|}/\frac{\left|NSD\cap IFD\right|}{\left|NSD\right|}$$

### GWAS data for diseases

We used GWAS data for coronary artery disease (CAD) and schizophrenia (SCZ), which were publicly available from the CARDIoGRAM consortium [29] and Psychiatric Genomic Consortium (PGC) [30], respectively. The CARDIoGRAM consortium performed a meta-analysis on 22 GWASs of individuals of European descent imputed by HapMap 2, which included 22,233 cases and 64,762 controls. The PGC included a multi-stage schizophrenia GWAS for 36,989 cases and 113,075 controls. From these GWASs, we found that 3,188 (out of 2,420,360) and 54,688 (out of 9,444,230) SNPs with moderate significance (10^−7^ < *p* ≤ 10^−3^) were associated with CAD and SCZ, respectively. We assigned each SNP to genes that were located within 10 kb of the gene (downstream or upstream), resulting in the assignment of 3,188 SNPs to 204 genes for CAD and 54,688 SNPs to 1,044 genes for SCZ. These genes were further filtered by the number of PSDs relevant to the diseases.

### Zebrafish housing and manipulation

Adult zebrafish were maintained at 28.5°C with a 13:11 h light:dark cycle in the Zebrafish Auto System (pH 7.0–7.9, Genomic-Design, Korea). Zebrafish embryos were collected after natural breeding and incubated in clean petri dishes with E3 medium (297.7 mM NaCl, 10.7 mM KCl, 26.1 mM CaCl_2_, and 24.1 mM MgCl_2_) containing 1% methylene blue (Sigma-Aldrich, St. Louis, MO, USA) at 28.5°C. For observation and photography, the embryos were raised (24 hours after fertilization) in the E3 medium containing 0.2 mM N-phenylthiourea (PTU; Sigma-Aldrich Chemistry, cat. # P7629) to block melanin formation.

### Microinjection with morpholino oligomers (MOs)

Translation-blocking MOs targeting coronary artery disease (CAD) candidate genes were designed and synthesized by Gene Tools (Philomath, OR 97370, USA). Each MO was diluted in distilled water at a concentration of 2 μg/μL and then injected into the yolk of zebrafish embryos at 1–4 cell stages using a gas-based microinjection system (Genomic-Design, Korea).

### Imaging the cardiovascular system of zebrafish

Overall morphology, heart asymmetry, and vascular phenotypes of the Tg(flk1:EGFP) zebrafish were observed using a fluorescent stereomicroscope (SMZ1270, Nikon, Tokyo, Japan). Images were captured using a camera (DS-Qi2, Nikon, Tokyo, Japan) and analyzed using NIS-Elements imaging software (Nikon, Tokyo, Japan).

## Supporting information

S1 Fig Regression analysis between Pathway Specificity (PS) and the log likelihood of human InterPro domains and GOBP pathways by InterPro2GO database. (TIF)

S2 Fig *In vivo* validation of candidate coronary artery disease (CAD) genes. **(a)** The sequence of translation-blocking MOs targeting candidate CAD genes used for this study. **(b)** Tg(*flk1*:*EGFP*) zebrafish embryos were injected with morpholinos (MOs) for candidate CAD genes and compared with control MO-injected embryos (morphants). The majority of morphants, except for *apod* morphants, exhibit gross morphological abnormalities, including a small brain, heart edema, and curved tail, at 3 days post-fertilization (scale bar = 500 μm). **(c)** Diagrams show the representative heart defects, such as no asymmetry (midline) and reversed asymmetry between ventricle and atrium, at 3 days post-fertilization.
(TIF)

S3 Fig Robustness of observed enrichment of PSD for disease-associated variants and for variants affecting physical protein interactions. In addition to the set of PSDs used for the analysis (4506 PSDs with 16k DPAs by PS threshold of 0.056), we also tested a smaller set of PSDs by more stringent PS threshold (0.066) resulting in 14k DPAs and 4341 PSDs and a larger one by more loose PS threshold (0.05) resulting in 18k DPAs and 4654 PSDs. We found that moderate changes in PS threshold for defining PSDs did not significantly affect enrichment of PSD for disease-associated variant by GWASdb (a) and for nonsynonymous variant affecting physical protein interactions by IMEx (b).
(TIF)

S1 Table The list of 49636 associations between 5253 human InterPro domains and 407 GOBP pathways with pathway-specificity (PS) scores. (XLSX)

S2 Table Pathway-specific domains for coronary artery disease (CAD). (XLSX)

S3 Table Pathways associated with coronary artery disease (CAD) (P < 0.01 by Fisher’s exact test and # pathway member genes > = 5). (XLSX)

S4 Table Pathway-specific domains for schizophrenia (SCZ). (XLSX)

S5 Table Pathways associated with schizophrenia (SCZ) (P < 0.01 by Fisher’s exact test and # pathway member genes > = 5). (XLSX)

S6 Table Priorized genes for coronary artery disease (CAD) by # of PSD (GWAS∩PSD set is highlighted). (XLSX)

S7 Table Priorized genes for schizophrenia (SCZ) by # of PSD (GWAS∩PSD set is highlighted). (XLSX)
