# Supplementary figures and images for: Pathway-specific protein domains are predictive for human diseases
Source: PLoS Comput Biol. 2019 May 10;15(5):e1007052. doi: 10.1371/journal.pcbi.1007052 (PMC6530867; doi:10.1371/journal.pcbi.1007052)

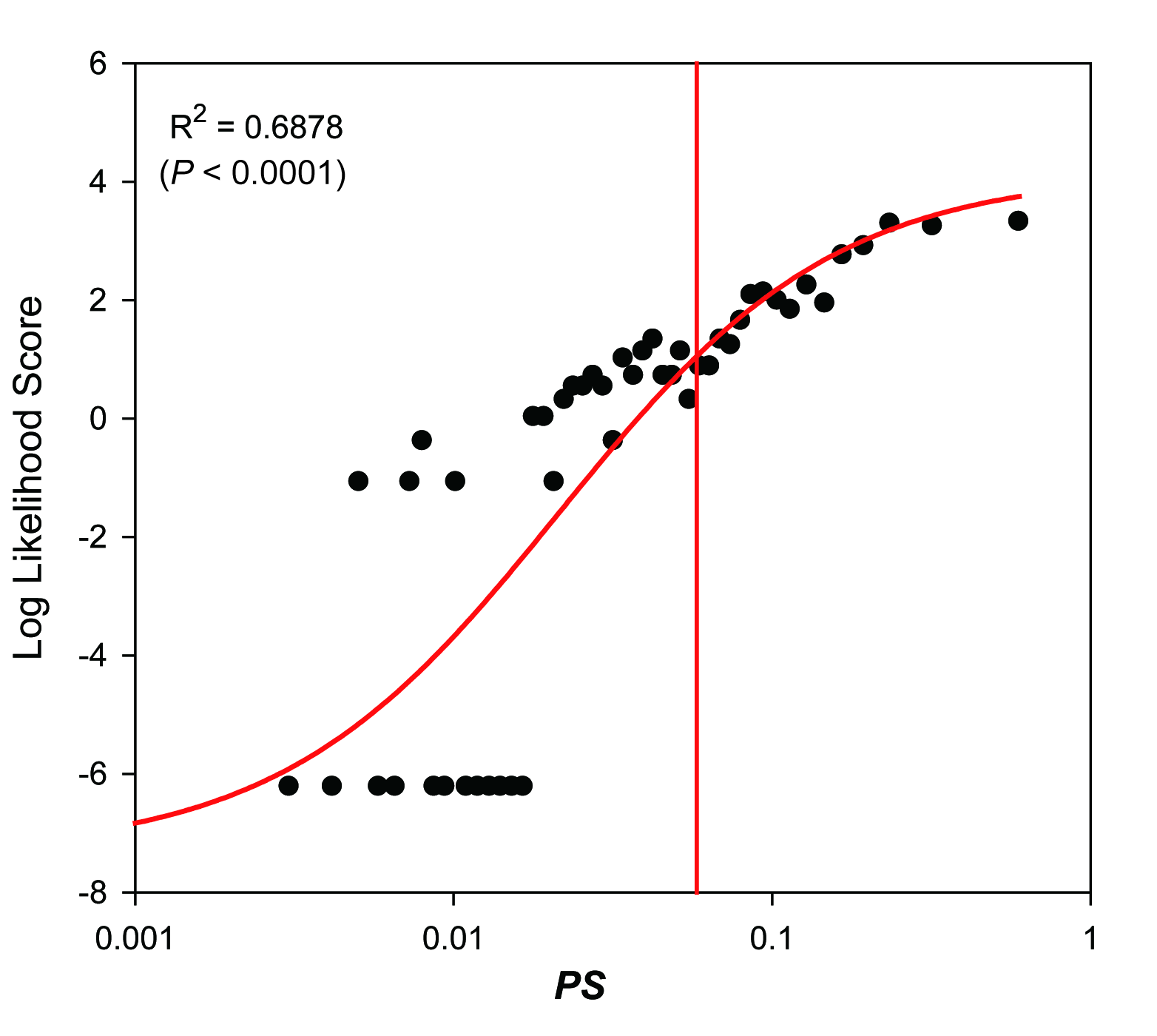

Supplement: S1 Fig — (TIF) [file pcbi.1007052.s001.tif]

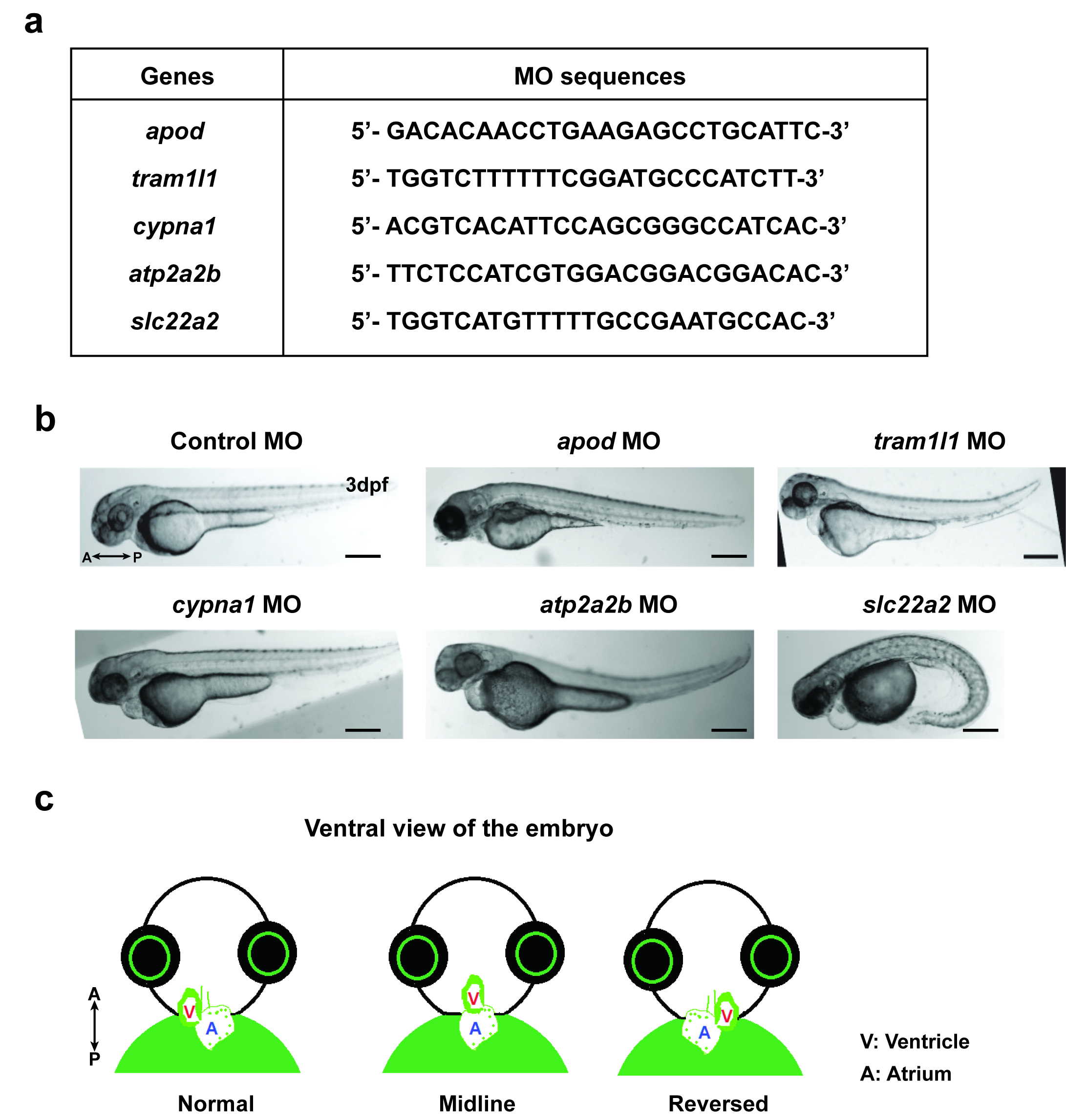

Supplement: S2 Fig — (a) The sequence of translation-blocking MOs targeting candidate CAD genes used for this study. (b) Tg(flk1:EGFP) zebrafish embryos were injected with morpholinos (MOs) for candidate CAD genes and compared with control MO-injected embryos (morphants). The majority of morphants, except for apod morphants, exhibit gross morphological abnormalities, including a small brain, heart edema, and curved tail, at 3 days post-fertilization (scale bar = 500 μm). (c) Diagrams show the representative heart defects, such as no asymmetry (midline) and reversed asymmetry between ventricle and atrium, at 3 days post-fertilization. (TIF) [file pcbi.1007052.s002.tif]

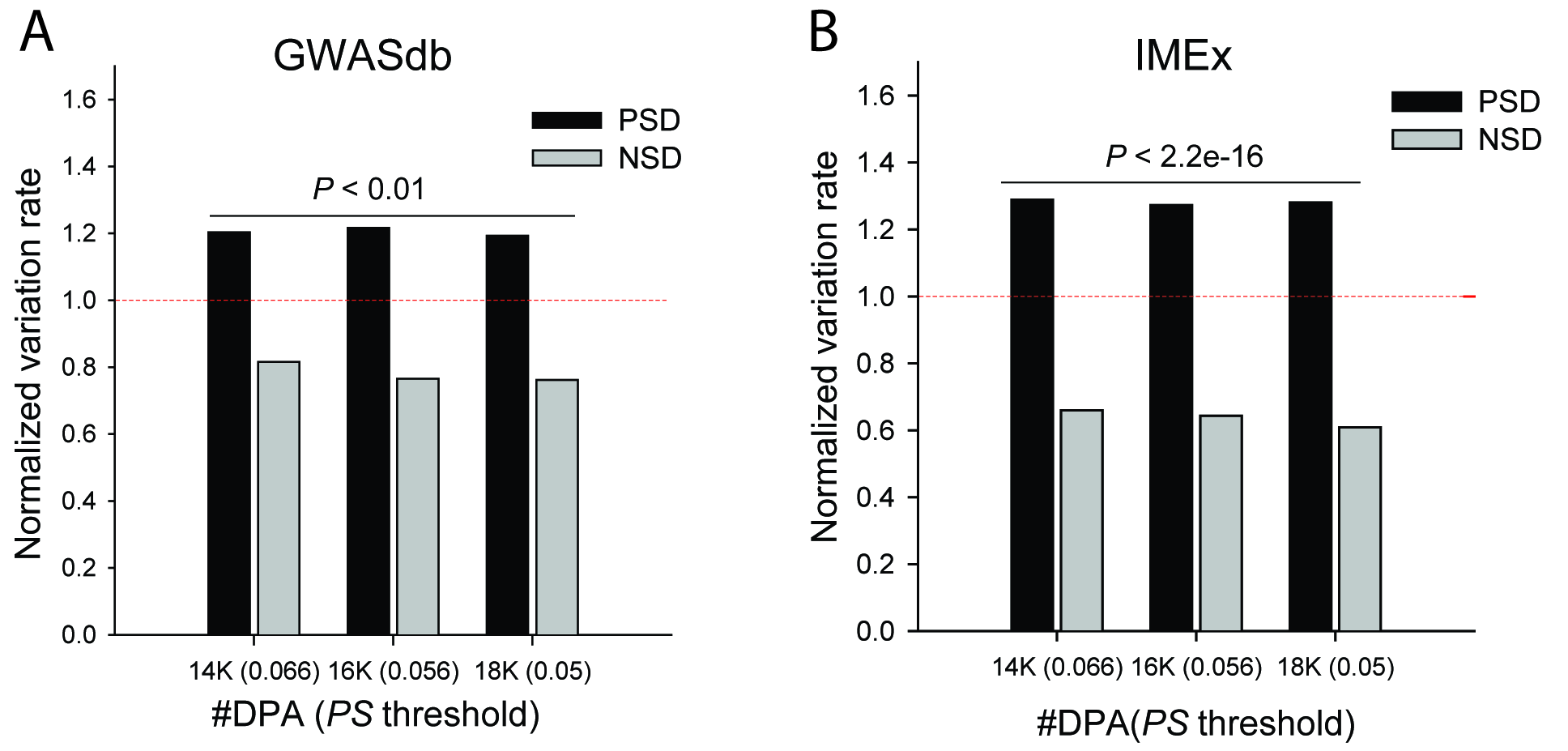

Supplement: S3 Fig — In addition to the set of PSDs used for the analysis (4506 PSDs with 16k DPAs by PS threshold of 0.056), we also tested a smaller set of PSDs by more stringent PS threshold (0.066) resulting in 14k DPAs and 4341 PSDs and a larger one by more loose PS threshold (0.05) resulting in 18k DPAs and 4654 PSDs. We found that moderate changes in PS threshold for defining PSDs did not significantly affect enrichment of PSD for disease-associated variant by GWASdb (a) and for nonsynonymous variant affecting physical protein interactions by IMEx (b). (TIF) [file pcbi.1007052.s003.tif]
